# Light-based 3D printing of mechanoluminescent living gels loaded with dinoflagellates

**DOI:** 10.1126/sciadv.adz0017

**Published:** 2026-06-05

**Authors:** Rani Boons, Mathias Steinacher, Henning Galinski, Vincent Niggel, Tanja Zimmermann, Gustav Nyström, Gilberto Siqueira, André R. Studart

**Affiliations:** ^1^ Complex Materials, Department of Materials, ETH Zurich, 8093 Zurich, Switzerland.; ^2^ Cellulose and Wood Materials Laboratory, Empa, Swiss Federal Laboratories for Materials Science and Technology, 8600 Dübendorf, Switzerland.; ^3^ Laboratory for Nanometallurgy, Department of Materials, ETH Zurich, 8093 Zurich, Switzerland.; ^4^ Laboratory for Soft Materials and Interfaces, Department of Materials, ETH Zurich, 8093 Zurich, Switzerland.; ^5^ Department of Health Sciences and Technology, ETH Zürich, 8092 Zürich, Switzerland.

## Abstract

Dinoflagellates, a group of marine unicellular algae, are known for the fascinating glowing effects in coastal waters. While this natural mechanoluminescent phenomenon has been explored in pressure sensors and optical transducers, technologies to shape dinoflagellate-containing materials into more complex, engineering-relevant geometries remain limited. Here, we report a three-dimensional printing strategy to manufacture complex-shaped mechanoluminescent objects using dinoflagellates embedded in biocompatible hydrogels. The growth and mechanoluminescence of the entrapped dinoflagellates were investigated by optical microscopy, emission spectroscopy, and mechanical testing of cell-laden gels. Dinoflagellate-laden gels showed strong bioluminescence when compressed at sufficiently high strain and strain rates. By incorporating the dinoflagellates into a photo-curable hydrogel, we shaped such living material into complex geometries using a widely available light-based printing technique. The ability to print dinoflagellate-laden gels into intricate shapes broadens the design space available for the creation of mechanoluminescent living objects for applications in soft robotics, self-powered sensing, and optical transduction.

## Teaser

Mechanoluminescent gels are produced by embedding dinoflagellates in complex-shaped structures using light-based 3D printing.

## INTRODUCTION

Engineered living materials harness the metabolic activity of microorganisms to imbue synthetic matter with previously unknown functionalities, such as self-healing, growth, self-regeneration, morphing, and adaptation (*1*–*8*). Besides these unusual properties, the metabolism of microorganisms also allows for the synthesis of materials in water at room temperature using environmentally friendly chemistries. The ability to genetically program the microorganisms to achieve specific functionalities is another unique feature of this emerging class of materials. Using a broad range of wild-type or engineered species, living materials have been exploited to create self-healing concrete (*9*–*11*), living pharmaceuticals (*12*), self-regenerating mycelia (*13*, *14*), hydrogel adhesives (*4*, *6*), living chemical sensors (*15*–*17*), and bioelectronics (*7*, *18*, *19*). Recently, the repertoire of microorganisms used in living materials has been extended to include mechanoluminescent dinoflagellates. By incorporating dinoflagellates into elastomers, biohybrids with fast, self-powered luminescence were created for soft robotic applications (*20*). This led to living materials that emit light under mechanical stress via a fundamentally distinct mechanism compared to existing mechanochromic and color-changing synthetic materials (*21*–*24*).

Dinoflagellates are marine unicellular algae known for their remarkable mechanoluminescence in oceans. While the biological function of such mechanoluminescence is still a matter of debate, the stress-triggered emission of light has been hypothesized to serve as a defense mechanism of the algae against predators. By producing flashes of blue light, dinoflagellates disrupt predator feeding and serve as a “burglar alarm” that visually attracts other secondary predators that prey on the initial attacker (*25*, *26*). The luminescence mechanism involves a stress-induced intracellular pH change that activates the enzyme luciferase to oxidize the substrate luciferin, thus generating an excited intermediate molecule that produces light when it decays from a high to a low energy state. Such a mechanism has been exploited for sensing of water impact pressure (*27*) and shear stresses (*28*). To create luminescent biohybrids, dinoflagellates were incorporated into chambers of preexisting silicone structures (*20*, *29*). This approach enables the fabrication of vibration-sensitive displays (*29*) and of soft robots that can visualize mechanical perturbations, illuminate a dark environment, and produce optical signals (*20*). Despite these remarkable capabilities, the fact that the dinoflagellates are not embedded directly within the elastomeric network makes them experience a stress that is not necessarily in correlation to the stress on the external material. Furthermore, full enclosure of the cells prevents feeding, unless the device is completely reopened and the suspension of dinoflagellates is exchanged, giving the material a finite life span. To fully exploit the potential of dinoflagellates in living materials and address these possible limitations, other manufacturing approaches need to be explored and developed.

Three-dimensional (3D) printing is an attractive manufacturing approach to create living materials with complex geometries and functionalities (*1*, *13*, *15*, *30*–*34*). Complex-shaped living materials with bioluminescent, regenerative, structural, chemical sensing, and bioremediation functionalities have been fabricated using extrusion- or light-based printing platforms. In these examples, wild-type or engineered microorganisms were loaded into hydrogels with rheological and photo-curing properties that enable 3D shaping of living materials into intricate geometries that do not exist in the natural world. Through 3D printing of living materials, it is possible to combine distinct microorganisms in the same structure with tuneable spatial control, to shape selected microorganisms into functional geometries not accessible via conventional manufacturing and to create sustainable organic, inorganic, or hybrid structures under mild processing conditions. By combining the shaping freedom provided by 3D printing with the rich metabolism offered by microorganisms, it is therefore possible to create materials with living properties thus far unavailable in synthetic systems. Recent research has demonstrated the potential of extrusion-based 3D printing for the fabrication of mechanoluminescent living composites using dinoflagellates embedded in hydrogels (*35*). Building on the impressive luminescence, mechanical properties, and extended lifetime of existing living composites (*35*), further development is required to fabricate living objects with tunable 3D geometries, predictable mechanoluminescent behavior, and application-tailored sensing capabilities.

Here, we develop and study hydrogels that are loaded with dinoflagellates to enable light-based 3D printing of complex-shaped living sensors with unique mechanoluminescent properties. The biological machinery underlying the mechanoluminescence of dinoflagellates is harnessed to create living objects that light up only when a critical mechanical strain or strain rate is reached. For this, we first grow dinoflagellates in model agar gels and study their mechanoluminescent response under different mechanical loading conditions. Next, we conduct time-resolved optical spectroscopy on single-cell colonies to better understand the physical processes underlying the mechanoluminescence of the living gels. The dinoflagellates are then embedded in light-curable hydrogels and printed into intricate 3D structures using the Digital Light Processing (DLP) technique. Last, we demonstrate how the strain-dependent luminescence of dinoflagellate-laden gels can be leveraged to print 3D living sensors that can detect forces that are relevant for manipulation tasks in robotic and prosthetic applications.

## RESULTS

Living materials with mechanoluminescent properties arising from dinoflagellates are prepared in two main steps: First, wild-type dinoflagellates are grown in liquid marine culture medium or embedded in soft hydrogels. The mechanical properties of these protective hydrogels need to be tuned to ensure effective entrapment of the microorganisms while also allowing for controlled cell proliferation to form metabolically active colonies. Second, hydrogels loaded with a high concentration of dinoflagellates are shaped into 3D structures using state-of-the-art DLP printing. This requires the design of hydrogels with rheological properties that meet the demands of this additive manufacturing technique.

*Pyrocystis lunula* was chosen as a model dinoflagellate species in this work because of its well-studied bioluminescence mechanism and its 10-fold higher bioluminescence compared to other species (*25*). Luminescence in dinoflagellates occurs in the scintillons, which are the light-emitting organelles of the cell. Light is produced from the chemical oxidation of the substrate luciferin in the presence of the enzyme luciferase. The oxidation product of this reaction, called oxyluciferin, emits a photon when the oxidized molecule decays from a high- to a low-energy state. Photon emission is triggered by external mechanical stimuli, which change the voltage across proton channels of the membrane and thereby reduce the pH from 8 to 6 inside the scintillons. Such a pH reduction increases the enzymatic activity of luciferase, leading to the rapid production of oxyluciferin that culminates with a flash of blue light at a peak wavelength of approximately 470 nm.

### Dinoflagellate responses in model agar gels

The growth of *P. lunula* in hydrogels was investigated by first using agar gels as a model medium with poro-viscoelastic properties comparable to those of printable formulations after cross-linking. On the basis of earlier work (*36*), we use hydrogels with a minimum agar concentration of 1%. With a low elastic modulus of 10 kPa and the ability to relax 90% of the internal stresses in less than 15 min, hydrogels with 1% agar should display the mechanical properties needed to promote the proliferation of microorganisms while at the same time minimizing cell leakage during growth. Gels were prepared by first dissolving agar in water by microwaving and cooling to 50°C, followed by casting the fluid into cold molds to enable solidification. To ensure cell viability, dinoflagellates were added and mixed into the fluid solution just before the casting step.

**Fig. 1.**
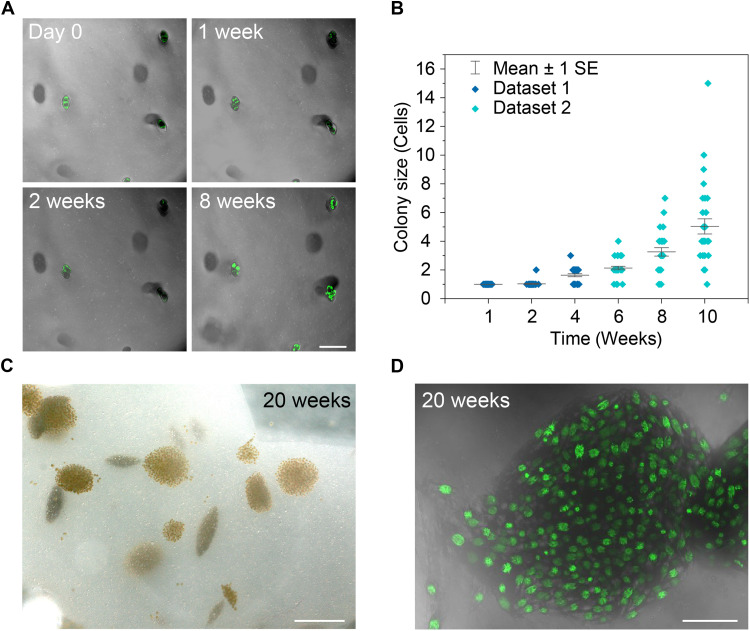
Growth and viability of *P. lunula* in 1% agar hydrogels. (**A**) Confocal microscopy images of dinoflagellate cells after growth in the agar gel for up to 8 weeks. The images were obtained from a 1150-μm-sized z-stack inside of the agar gels. The green channel of the microscope was used to visualize the cells through the autofluorescence of chloroplasts inside of the dinoflagellates. Scale bar, 250 μm. (**B**) Growth curve displaying the number of cells contained in entrapped colonies over time (*n* = 30). (**C**) Bright-field image and (**D**) z-stack confocal images of dinoflagellate colonies entrapped in the gel after 20 weeks of incubation. Scale bars, 1 mm (C) and 250 μm (D).

Cell proliferation was quantified by tracking the number of dinoflagellates in individual colonies as a function of time (Fig. 1). To this end, we take advantage of the autofluorescence of the chloroplasts to facilitate the visualization of metabolically active algae cells (Fig. 1A). Our growth experiments show that the number of dinoflagellates per colony increases from one to an average of five cells over a period of 10 weeks (Fig. 1B). The algae colonies obtained after 10 weeks vary broadly in cell numbers from 1 single cell to as many as 15 cells.

Proliferation in the agar gel was found to be much slower than that reported in liquid media, in which dinoflagellates typically double in about 2 weeks (*26*, *37*). This might arise from confining residual stresses induced in the gel after cell division, as previously shown for diatoms growing in comparable agar samples (*36*). Despite the slower growth in gels, our experiments show that dinoflagellates were able to proliferate successfully and at a similar rate in hydrogels with 0.5, 1, 2, and 3% agar. Culturing of *P. lunula* for 20 weeks allowed for the preparation of agar hydrogels with a high density of colonies up to 1 mm in size (Fig. 1, C and D). Such population of large colonies are readily visible by optical microscopy and therefore suitable for the generation of strong bioluminescence when subjected to mechanical stimulus.

**Fig. 2.**
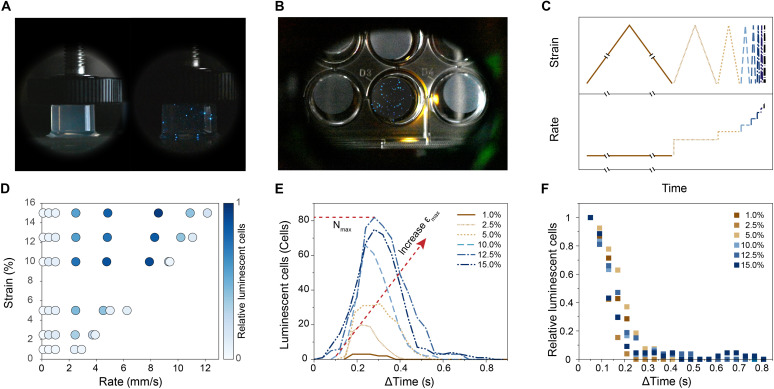
Mechanoluminescent response of agar gels loaded with dinoflagellates. (**A**) Photographs of cylindrical samples subjected to uniaxial compression. The 10 mm–by–12 mm samples contain *P. lunula* cells at a density of 1 cell/μl. The left image shows the hydrogel before compression, whereas the image on the right features the mechanoluminescence produced upon compression of the gel at 9.3 mm/s in low-illumination conditions. (**B**) Image from gels compressed at 2.5 mm/s and 10% strain acquired from the bottom of the sample. This configuration was used to quantify luminescence via image analysis. The setup was partly illuminated while keeping the compressed gel in the dark. (**C**) Schematic of strains and strain rates imposed on the samples during cyclic mechanical testing. (**D**) Color map displaying the number of cells that generate luminescence in the cell-laden gels when subjected to compression cycles at different strains and strain rates. The color intensity was normalized by the maximal number of emitting cells in the samples during compression. Data were obtained from six cyclic tests, in which the strain rate was progressively increased [see plot (C)], while keeping the applied strain constant (*n* = 4). (**E**) Number of luminescent cells over time for gels compressed using distinct maximum strains ($\varepsilon_{\text{max}}$) at a constant strain rate of 2.5 mm/s. *N*_max_ indicates the maximum number of luminescent cells. (**F**) Decay of the relative number of luminescent cells over time for samples subjected to strains of 1 to 15% at a strain rate of 2.5 mm/s.

The bioluminescence of dinoflagellates entrapped in agar gels was investigated by conducting compression experiments on cylindrical samples freshly prepared from cell cultures incubated for 4 days. We quantified the bioluminescence by performing mechanical tests in a customized setup that allows for direct imaging from the side and the bottom of the sample during uniaxial compression (Fig. 2, A and B). First, qualitative experiments showed that the dinoflagellates emit intense blue light upon compression of the agar gel. This demonstrates that the entrapped cells are metabolically active and that the externally applied stresses can be effectively transferred through the gel to trigger the bioluminescent response of the dinoflagellates.

To explore the effect of mechanical stresses on the luminescent behavior of the algae-laden gel, we carried out mechanical tests in which a cylindrical sample was loaded and unloaded multiple times with progressively increasing strain rates while keeping the maximum strain constant, as schematically shown in Fig. 2C. Since the mechanical testing machine is not designed to operate at high speeds, we use calibration curves to determine the effective strain rates applied to the samples (fig. S1A). For low strains and high strain rates, the effective strain rates differ substantially from the nominal strain rates. Thus, the effective strain rate was calculated from the experimentally measured traveling distance and elapsed time. Following this approach, we were able to cover maximum applied strains ($\varepsilon_{\text{max}}$) in the range 1 to 15% and strain rates varying from 0.10 to 9.37 mm/s. Such a protocol was repeated with other samples by applying distinct levels of maximum strain to systematically characterize the mechanoluminescent response of the cell-laden gels.

Notably, our experiments revealed that a minimum strain and strain rate are required to activate the scintillons of the dinoflagellates and thus emit light (Fig. 2D and fig. S1B). Only above these thresholds, the mechanical forces generated in the gel are high enough to trigger the mechanoluminescent effect. These observations are in line with previous research on dinoflagellates in water, which suggests that the viscoelastic nature of the cells makes both the level of stress and speed of stimulation critical factors for the biological function of bioluminescence (*26*, *38*, *39*). By activating the luminescence response only above a certain stress and stimulation speed, the dinoflagellates make sure that this energetic process is only triggered under life-threatening events, such as the direct contact with predators (*26*). Although such a minimum triggering force has been confirmed by single-cell experiments (*26*), previous research on dinoflagellate-laden composites reported a continuous increase in luminescence with increasing strain rates (*35*). The presence of a threshold rate opens previously unidentified opportunities for the use of these living gels as mechanoluminescent sensors. By detecting strain rates only above an application-relevant threshold rate, such living sensor would enable decentralized decision-making with minimal power consumption (*40*).

Closer examination of gels loaded above the threshold rate allowed us to gain further insights into the evolution of the light emitted by the stimulated dinoflagellate cells. For samples compressed at the strain rate of 2.5 mm/s, we observed a monotonic increase of the number of luminescent cells over time until a peak is reached (Fig. 2E). This is followed by a continuous drop in the number of light-emitting cells. An increase in the $\varepsilon_{\text{max}}$ at a fixed strain rate was found to enhance the maximal number of activated cells ($N_{\text{max}}$) as well as the total amount of luminescent energy generated during compression, which should be proportional to the area under the curve displaying the number of luminescent cells versus elapsed time. This effect was observed for a broad range of strain rates. Moreover, our data indicate that the luminescence decay occurs within a timescale of 0.5 to 0.8 s, which allows for easy visualization by the naked eye and is comparable to the range of 0.2 to 0.6 s reported in the literature for stimulated dinoflagellates in water (*27*).

Besides the timescale of the emission decay, we can also correlate the imposed mechanical stimulus and the number of luminescent cells by estimating the level of stress exerted on the dinoflagellates during the compression tests. To estimate the compressive stresses necessary to trigger the luminescence of entrapped dinoflagellates, we first evaluated the stress-strain response of the gel samples during mechanical loading (fig. S2A). Representative stress-strain data obtained for a strain rate of 2.5 mm/s indicate that the agar gel displays a fully reversible mechanical response with a hysteresis behavior that is characteristic of polymeric hydrogels. A direct correlation was observed between the maximum strain $\left(\varepsilon_{\text{max}}\right)$ and the maximum stress ($\sigma_{\text{max}}$) developed in the hydrogel upon uniaxial compression.

To evaluate the effect of the maximum stress $\left(\sigma_{\text{max}}\right)$ on the number of luminescent cells, we select data obtained at the strain rate of 2.5 mm/s. This condition ensured that the chemical resources needed for bioluminescence were not yet consumed in the cyclic experiment since this strain rate lies right above the threshold needed to stimulate the cells (Fig. 2D). As expected from the stress-strain dependency, our analysis reveals that the maximum number of emitting cells ($N_{\max}$) increases when the maximum global stress is increased up to 2 kPa (fig. S1, C and D, and S2B). This result agrees with an earlier study on dinoflagellates stimulated in water, which reports a linear increase of bioluminescence for the stress range 0.1 to 1.2 kPa (*27*). We found that the number of light-emitting cells remains approximately stable above 2 kPa, indicating that there is a limit in the number of live dinoflagellates in the population that can be mechanically activated. The effect of global stress on the bioluminescence was observed to follow a similar trend for samples tested at the strain rates of 4.8 and 7.9 mm/s (fig. S2C). These two higher rates activate more cells (fig. S1, C and D), which suggests that the population of dinoflagellates comprises cells and colonies with different sensitivities to strain rate. For the higher rates above 9.3 mm/s, only weak luminescence was measured probably because of the exhaustion of the chemical resources the cell needs to emit light.

The exhaustion of chemical resources upon multiple stimulation events is evident when the cell-laden hydrogel is subjected to several consecutive compression cycles at fixed strain and strain rate (fig. S3 and movie S1). Upon the first compression, the cells respond with a strong flash, in line with earlier studies. Further compression cycles reduce the luminescence to below 10% of the intensity generated after the first event (fig. S3B) (*26*). A similar effect is observed for samples tested at higher strain rates.

To evaluate the effect of the strain rate on the bioluminescence in the presence of abundant chemical resources, we performed an additional series of experiments in which initially fresh cell-laden hydrogels are compressed only once at a predefined strain and strain rate (fig. S4). Since it is performed on fresh samples, this experiment ensures high availability of resources. The results reveal a continuous increase in the number of luminescent cells with the increase in the applied strain rate. The observed correlation between bioluminescence and the globally applied deformation rate indicates that the gel effectively translates this external mechanical stimulus into the local strains and strain rates required to trigger the dinoflagellate cells (*39*). Additional experiments show that the gels need to be sufficiently stiff to be able to translate the global stimulus into high local strain and strain rates. Because of their stiffer nature, hydrogels with higher agar concentrations lead to stronger bioluminescence (fig. S5). The bioluminescent dinoflagellates were found to remain alive and biologically active even after 20 weeks of incubation (Fig. 1D and movie S2).

### Spectral analysis of single dinoflagellate colonies

**Fig. 3.**
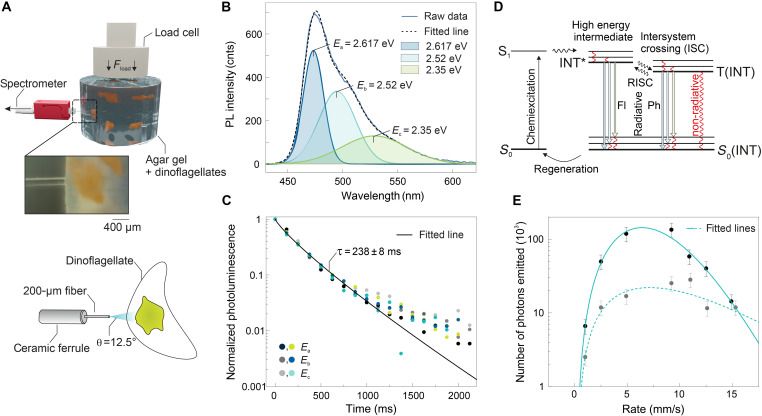
Mechanoluminescence spectroscopy on single dinoflagellate colonies. (**A**) Illustration of the experimental approach. Cylindrical hydrogels with a low density of *P. lunula* colonies are subjected to an axial load $F_{\text{load}}$. The time-dependent photoluminescence of single *P. lunula* colonies is probed using a fiber optic cannula that is inserted into the hydrogel before loading, as shown in the micrograph (inset). The cannula’s low angular acceptance θ for incoming light facilitates spectroscopic measurements of a single colony. (**B**) Time-integrated photoluminescence (PL) spectrum for a single *P. lunula* colony and Gaussian peak deconvolution that reproduces the measured spectrum. Characteristic emission was detected at three peak energies: *E*_a_ = 2.617 ± 0.007 eV, *E*_b_ = 2.51 ± 0.02 eV, and *E*_c_ = 2.35 ± 0.05 eV. (**C**) Energy-resolved time-dependent photoluminescence of two representative colonies. The measured data were fitted using a stretched exponential exhibiting a characteristic decay time τ = 238 ± 8 ms. (**D**) Partial energy diagram for the bioluminescence of *P. lunula*, indicating the mechanochemical excitation to an excited state ($S_{0}$→ $S_{1}$) and subsequent chemical conversion to a high energy intermediate ($S_{1}$ → INT*), and decay via radiative [fluorescence (Fl) or phosphorescence (Ph)] and nonradiative processes to a new ground state [$S_{0}$(INT)] with three vibrational bands possibly via (reverse) intersystem crossing (R)ISC. Last, a slow metabolic regeneration [$S_{0}$(INT) → $S_{0}$] takes place. (**E**) Number of photons emitted from single colonies as a function of the strain rate applied in consecutive cycles. The data points refer to a set of consecutive measurements with increasing strain rate performed on two distinct cell colonies. Depicted lines correspond to fitting of the derived logistic growth model to the experimental data (see the Supplementary Materials).

While the strain-induced emission of visible light offers an accessible readout for a force sensor, detailed optical analysis is expected to provide insights into the precision and nature of the bioluminescent signal. In this context, we took advantage of the immobilized state of dinoflagellates to measure the energy and number of photons emitted by single colonies after mechanical loading the host gel. This was accomplished by detecting the energy-resolved time-dependent photoluminescence of single colonies in a customized setup (Fig. 3A). Compared to earlier work on macroscopic samples (*35*), such an approach provides microscopic understanding of the mechanoluminescent response of dinoflagellates embedded in soft gels. Our experiments reveal that the measured time-integrated photoemission spectrum (Fig. 3B) displays three emission peaks (blue, cyan, and green) with decreasing photon energy, matching the turquoise color appearance observed visually. The peak photon energies are strain-rate invariant and prove to be statistically robust in-between colonies (fig. S6).

Analyzing the time evolution of the deconvoluted peaks provides more information on the microscopic mechanisms of bioluminescence in dinoflagellate-laden gels (Fig. 3C). This offers deeper insight that was not accessible by luminescence measurements on macroscopic composites (*35*). To illustrate this, we picked two colonies and measured the normalized time-resolved photoluminescence of all three emission peaks independently with a resolution of 125 ms. The time-dependent photoluminescence spectra are fitted by a Kohlrausch function (Supplementary Materials) to account for a continuous distribution of decay times that contribute to relaxation of the emitter. Curves for all measured emission peaks and colonies (Fig. 3C) are well represented by using an average lifetime of τ = 238 ± 8 ms and a stretch parameter α = 0.85. The measured lifetime is on the same order of magnitude of that observed for firefly luminescence, which also relies on luciferin to luminesce (*41*, *42*). However, the lifetime in the range of milliseconds substantially differs from time-resolved pump experiments of synthetic firefly luciferin in solution, where lifetimes of 40 to 400 ps have been determined (*43*). We attribute the difference in lifetime to our experimental setup, where we probe the average decay of an embedded biological system, while pump probe experiments exclusively probe the decay of the purified emitting molecule sourced from a different organism.

To interpret these experimental observations, we can construct a partial energy diagram of the bioluminescent states of dinoflagellates on a single-colony level (Fig. 3D). Triggered by mechanical forces (*39*, *44*), the luciferin in the dinoflagellates is excited from a ground state $S_{0}$ to an excited state INT*. While the details of the chemical process and the intermediate molecule are unknown, it is hypothesized that the formation of the excited state INT* involves the oxidation of the substrate luciferin by the enzyme luciferase to form oxyluciferin (*44*). The light-emitting state can decay from INT* to its ground state $S_{0}$(INT) by a singlet-singlet transition (fluorescence) or from T(INT) to the ground state $S_{0}$(INT) by a triplet-singlet (phosphorescence) relaxation (*45*). The measured, relatively large average lifetime τ in the order of hundred milliseconds suggests the presence of a rather slow process step during biochemical conversion and radiative decay to three distinct vibrational energy levels of the electronic ground state. The energy level splitting of the observed states (Fig. 3B) corresponds to wave numbers between 900 and 1269 cm^−1^, which is characteristic of vibrational states.

The outlined decay to a “new” ground state $S_{0}(INT)$ that is chemically different than $S_{0}$ leads to a depletion of the luciferin pool, which can be observed when executing a series of measurements with increasing strain rate (Fig. 3E). The number of photons initially increases with increasing strain rate, but because of the depletion of the reservoir of emitters, the trend is reversed and the signal decays at the highest tested rates. We can describe this behavior by constructing a logistic growth model considering both the positive correlation between strain rate and bioluminescence and the exhaustion of the luciferin pool (see Supplementary Materials).

### Light-based printing of bioluminescent gels

The mechanoluminescence of entrapped dinoflagellates can be harnessed to create functional living objects by incorporating *P. lunula* cells in 3D printable hydrogel inks. To explore this idea, we designed a light-curable, biocompatible hydrogel that displays mechanical properties similar to agar gels and that can be printed into complex geometries using a desktop DLP printer. Following a previously reported formulation (*15*), the hydrogel ink comprises 1% of methacrylated hyaluronic acid (HAMA) and 20% of a dimethacrylated PEO-PPO-PEO copolymer (Plu-DMA) in marine culture medium. These reactive species are complemented with 0.5% of the photoinitiator lithium phenyl-2,4,6-trimethylbenzoylphosphinate (LAP) to initiate the photo-polymerization and cross-linking reactions. The dye quinoline yellow (0.018%) was also added to the ink to control light penetration into the hydrogel during the printing process.

To evaluate whether the light-curable hydrogels are also able to trigger the luminescence of embedded dinoflagellates, we fabricated cylindrical samples and analyzed their mechanoluminescent response under selected strain rates (fig. S7). The results show that a strain rate of 0.5 mm/s is not sufficient to activate the embedded cells. Bioluminescence is only observed when samples are compressed at an increased strain rate of 2.5 mm/s. We therefore conclude that dinoflagellates embedded in gels require a critical strain rate to bioluminesce, independent of the exact composition of the host matrix. In soft robotics, this threshold strain rate can be engineered to act as a band-pass filter, suppressing background noise while selectively detecting force signals that are relevant for robotic function. Comparable to the distributed sensing found in octopus arms (*46*) and in the Venus fly trap (*47*), this approach enables decentralized decision-making and alleviates the need for energy-intensive data processing in the central control unit (the “brain”). By using light as input, such a soft living sensor also avoids the complexity of electric wiring and the stiffening effect of electrically conductive fillers while exploiting the high speed of optical signal transmission (*48*).

**Fig. 4.**
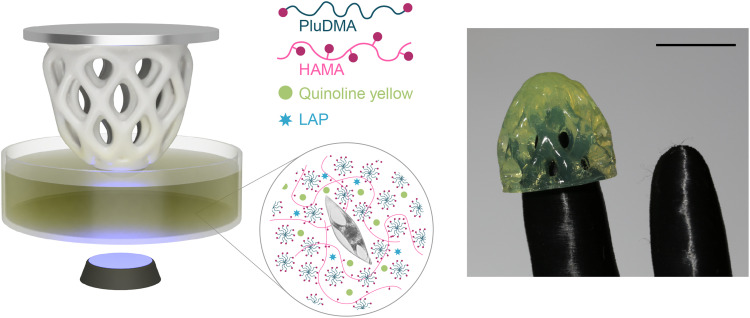
DLP printing of hydrogel objects loaded with dinoflagellates. (**Left**) Cartoon of the light-based printer setup, highlighting the formulation of the photo-curable ink containing the dinoflagellates. The ink is composed of the two photoreactive polymers PluDMA and HAMA, the biocompatible photoinititator LAP, the dye quinolone yellow, and the *P. lunula* cells. (**Right**) 3D-printed porous cap for a robotic fingertip. Scale bar, 1 cm.

Complex-shaped living objects were created by DLP-printing the light-curable hydrogel inks in a layer-by-layer fashion using a commercially available printer equipped with an ultraviolet light source at 405 nm (Fig. 4). Expanding on the predominantly planar patterns generated with a previously reported extrudable ink (*35*), the DLP setup allows printing of complex geometries relevant for soft robotic applications. To illustrate this, we printed a finger cap with an open cell architecture that allows physical contact of the soft gel with the surrounding environment while increasing the availability of the oxygen necessary for the metabolic activity of the embedded dinoflagellates (Fig. 4).

**Fig. 5.**
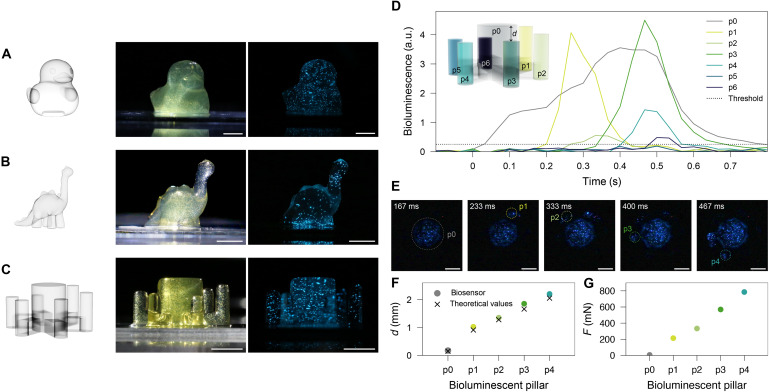
DLP printing of mechanoluminescent living objects using hydrogels loaded with dinoflagellates. (**A** to **C**) CAD model (left), images of 3D-printed hydrogels containing living dinoflagellates (middle) and mechanoluminescent living hydrogels emitting blue light because of a manual impact on the table where they are placed (right). Scale bars, 5 mm. (**D**) Temporal evolution of the bioluminescence for each pillar of the force biosensor under compression. (**E**) Snapshots of the compressed force biosensor imaged from below showing the sequential light up of the sensor pillars. Scale bars, 2 mm. (**F**) Compression distance *d* required to stimulate the bioluminescence in each pillar and the corresponding theoretical distance values from the sensor design. (**G**) Compressive force required to trigger the bioluminescence of the sensor pillars.

Two other illustrative geometries were selected to further demonstrate the printing process: a duck- and dinosaur-shaped monolith (Fig. 5, A and B) and an open gyroid structure (fig. S8). In contrast to agar gels, the hydrogel inks are sufficiently fluid at room temperature to easily replenish the printing tray with a new reactive layer after each sequential illumination step (*15*). After photo-curing, the elastic modulus of the cross-linked hydrogel (16 ± 1 kPa) are on the same order of magnitude of the previously used model agar gels (22 ± 3 kPa) (fig. S9) (*36*).

Our experiments show that the centimeter-sized duck-, dinosaur- and gyroid-shaped objects could be successfully printed using the hydrogel inks loaded with a high concentration of dinoflagellates (Fig. 5, A and B, and S10). In addition to the effective entrapment of the dinoflagellate cells, the cross-linked hydrogel was found to be sufficiently stiff to prevent gravity-induced distortions of the printed objects. Cells entrapped in the printed gels also retained their shape and showed a dark brown core that is characteristic of living dinoflagellates (fig. S10). The cells remained metabolically active in the biocompatible hydrogels. To validate this, we manually hit the table where the printed objects were placed in the dark to generate a strong and fast mechanical stimulus. The objects immediately glowed in response to the imposed impact (Fig. 5, A and B, and movie S4), thus demonstrating the possibility to effectively entrap dinoflagellates in a biocompatible gel and print the resulting soft material into mechanoluminescent living objects of complex shapes. In addition to DLP, we also developed an extrusion-based 3D bioprinting to create mechanoluminescent structures from gels loaded with living dinoflagellates (fig. S11).

The ability of the dinoflagellate-laden gels to transduce a mechanical stimulus into a macroscopically observable optical signal was harnessed to generate a functional living device for stress sensing (Fig. 5C). While the shaping freedom of DLP-printing allows for the manufacturing of application-relevant complex geometries, we selected a simple geometrical design to illustrate the concept of the sensor. The sensor consists of a circular main pillar (p0) and six shorter pillars (p1 to p6) of decreasing heights (h) arranged around the central pillar (Fig. 5D, inset). In this design, the decreasing heights of pillars p1 to p6 ($h_{p1}>h_{p2}>h_{p3}>h_{p4}>h_{p5}>h_{p6}$) make them luminesce in a sequential manner when the sensor is mechanically pushed by a flat puncher from the top. Pillar p0 displays a larger diameter that provides mechanical stability to the sensor, whereas pillars p1 to p6 serve as discrete force indicators through bioluminescence upon stimulation.

We experimentally demonstrate the working principle of the force-biosensor by compressing the whole pillar construct in a mechanical testing machine. For this, the sensor was compressed by 38.5% strain at a rate of 5 mm/s while recording the applied force and imaging the bioluminescence from below (movie S5). The bioluminescence emitted by the distinct pillars was measured as a function of time. The results confirmed the sequential lighting of the five largest pillars (p0 to p4), as expected from their initial heights (Fig. 5, D and E, and movie S5). The shortest pillars (p5 and p6) did only partially light up and were not considered for further analysis. To evaluate whether the emitted light signal correlates with the applied input strain, we compared the nominal displacement imposed by the testing machine with the displacement estimated from the measured bioluminescence of each pillar.

Our analysis shows that the pillars light up at the displacements expected from the geometry of the sensor (Fig. 5F). Using the measured force-displacement data (fig. S12), the living device can also be used to detect imposed compressive forces (Fig. 5G). For this specific geometry, the imposed force range can be assessed from the bioluminescent signals of the individual pillars. If only pillar p1 lights up, the applied force lies between 214 and 333 mN. Instead, if both pillars p1 and p2 luminesce, the force ranges from 333 to 569 mN. We envision this type of living sensor to be used, for example, on the tip of a soft robotic arm to optically indicate the force applied on the robot upon contact with surrounding objects. With a diameter of 1.6 mm, the individual pillars are comparable to tactile pixels (taxels) used in robotics and prosthetics (*49*). Moreover, the detectable force range of 0.1 to 1 N falls within the force window typically required for gentle manipulation and grasping tasks (*50*).

## DISCUSSION

The mechanoluminescent printed structures may be regarded as synthetic functional life-forms that embody the dinoflagellates in an environment that is very distinct from their natural marine habitat. In contrast to conventional materials, the living mechanoluminescent object is self-powered by the chemical energy provided by nutrients, which can potentially be harnessed from the environment. Although the resources for bioluminescence deplete when repeatedly actuated, the living cells recharge through their metabolism. Moreover, the fast response time in the order of a few hundred milliseconds or lower (*51*) is compatible with state-of-the-art transducing technologies. Notably, the dinoflagellate-laden material can be printed in water at room temperature to generate complex-shaped living objects using fully biocompatible ingredients. Combined, the metabolic activity of dinoflagellates and the shaping freedom of 3D printing provide an enticing approach to design and fabricate mechanoluminescent living materials for optics-based mechanosensing, signaling, illumination, and soft robotic applications.

## MATERIALS AND METHODS

### Materials

Chemicals were used as received unless stated otherwise. Agarose (Agarose Low Melting, Lot AS489940) was obtained from Apollo Scientific; hyaluronic acid (sodium hyaluronate, 95%) was purchased from Acros Organics; while calcium chloride (CaCl_2_ ≥ 96%), Dulbecco’s phosphate-buffered saline (DPBS) solution, quinoline yellow, lithium phenyl-2,4,6-trimethylbenzoylphosphinate LAP (≥ 95%), methacrylic anhydride (94%), Pluronic F-127, diethylether (≥ 99.8%), hydrochloric acid (37%), and sodium hydroxide pellets (98 %) were supplied by Sigma-Aldrich. Last, cellulose nanocrystals (CNC) prepared via sulphuric acid hydrolysis of bleached wood pulp were purchased from CelluForce.

### Synthesis of HAMA and Plu-DMA

HAMA was prepared following a previously described protocol (*15*, *52*). In a 250-ml round-bottom flask, 1 equiv (1 g) of sodium hyaluronate was dissolved overnight into 100 ml of deioinized (Milli-Q) water. The pH was kept at 8 by dropwise addition of NaOH solution (1 M). Subsequently, the solution was cooled down in an ice bath after which 20 equiv (7.86 ml) of methacrylic anhydride was added. While vigorously magnetic stirring, the pH was adjusted to 8 by adding NaOH for a period of 3 hours. The reaction was continued overnight at 5°C under stirring conditions to reach a total reaction time of 24 hours. Next, the solution was precipitated in cooled ethanol, washed and dissolved again in Milli-Q water before being transferred to dialysis tubes (VWR, cut off 12 to 14 kDa). After dialysis purification for 5 days, the HAMA was freeze-dried and stored at −20°C until use.

To synthesize Plu-DMA, Pluronic F-127 was first dried at 40°C and 10 mbar as described elsewhere (*15*, *53*). Next, 100 ml of chloroform was added into a 250-ml round-bottom flask containing 1 equiv (50 g) of the dried Pluronic. The resulting solution was stirred magnetically until full dissolution of the polymer and cooled down in an ice bath. While stirring, the cooled solution was supplemented with 8 equiv (4.40 ml) of trimethylamine (TEA). After dissolution of the TEA, 8 equiv (4.73 ml) of methacrylic anhydride was added. The flask was flushed with dry nitrogen for 2 min, after which it was closed with a septum pierced by a needle connecting the flask to an argon-filled balloon. After 8 hours, the ice bath was removed and the reaction continued for additional 16 hours at room temperature. The polymer was collected using a Büchner filter after precipitation in cold diethyl ether and drying at 10 mbar. The overnight dried polymer was dissolved in CHCl_3_, mixed with cold diethyl ether in a 1:10 volume ratio, and centrifuged at 3350*g* to obtain the final Plu-DMA as a pellet after decanting the supernatant. The pellet was dried at 10 mbar overnight and stored at −20°C until further use.

### Algae cultures

*P. lunula* (CCAC3471B) purchased from the Culture Collection of Cologne (CCAC) was grown statically in sterile L1 medium (*54*) using T25 and T75 culture flasks or glass Erlenmeyer. The culture was subjected to a light/dark cycle of 12/12 hours. To study bioluminescence during the day, the dark cycle started at 8 to 9 p.m. central European time, depending on summer or winter clock. A mixture of red and blue light-emitting diode (LED) lamps (LED Plant Grow Panel Lamp System, HQRP) was used as light source. The heat produced by the light causes the temperature to fluctuate between 20° and 25°C. In addition to the cultures, agar-encapsulated dinoflagellates also served as an abundant source of dinoflagellates. Every 3rd week, the cultures were maintained by adding a volume of 10 to 50% fresh medium, depending on the density of the respective culture. Cultures reached on average densities of 0.1 to 1 cells/μl. By centrifuging the cells at 2000*g* for 10 min and removing the supernatant, the dinoflagellates could be collected and up-concentrated for further experiments. Cultivation and handling of dinoflagellates until included in the gel was always performed under sterile conditions.

### Visualization and counting of dinoflagellate cells

Dinoflagellates were visualized by exploiting the autofluorescence of the chlorophyll molecules naturally present in photosynthetic organisms. For fluorescent imaging, a confocal microscope (TCS SP8, Leica Microsystems CMS GmbH, Germany) was used using excitation and emission wavelengths of 538 and 650 to 700 nm, respectively. The lookup table (LUT) of the fluorescent image was chosen to indicate the chlorophyll in the chloroplasts. Digital (Digital VHX-7000 Keyence microscope, USA) and inverted light microscopes (inverted Phase Contrast Microscope, Leica Microsystems CMS GmbH, Germany) were also used to obtain images with natural colors. For recording and imaging bioluminescence, a digital camera (D5600 Digital SLR Camera, Nikon, Tokyo) equipped with a macro lens (AF-S VR Micro-NIKKOR 105 mm f/2.8G IF-ED, Nikon, Tokyo) was installed in a dark room or in front of a fully sealed cardboard box. Videos and images were captured using the following settings: f/2 = 1/30 F5 ISO = 16,000 and 25 to 30 fps. Cell counting was performed with the help of a fluorometric imager (ChemiDoc MP, BioRad Laboratories, Hercules, California), using an excitation wavelength of 650 nm for 0.2 s and a filter cube of 700/50 nm for detection. For counting, three aliquots of 100 μl of a dinoflagellate culture were placed on glass slides covered with a cover slide. Cells in the form of white dots could be easily detected by eye. Particle tracking codes were used to calculate the cell density of the respective culture.

### Mold casting of agarose hydrogels

Agar stocks of 1 to 6% w/v were prepared by mixing the low melting agarose in L1 medium and heating the mixture in a microwave at low power until the agar was fully dissolved. Stocks gelled at room temperature were reheated in a hot water bath at 70°C until liquid. To prepare the cell-laden hydrogels, the hot agar stocks were kept in a water bath at 50°C while preparing the dinoflagellate suspension. Dinoflagellates were collected as described above. The volume of culture medium in the suspension was controlled to reach a final density of 1 cell/μl in the hydrogels. On the basis of preliminary centrifugation experiments, it is assumed that 20% of cells are lost during collection. Next, the dinoflagellate suspension kept at room temperature was mixed in a 1:1 volume ratio with the 50°C agar stock using a 5- to 10-ml volumetric pipette. This procedure was found to keep the dinoflagellates alive and functional despite the short exposure to the warm agar solution. The mixture was placed in a mold of desired shape. After 2 to 5 min of gelling at room temperature, the hydrogels were removed from the mold and placed in fresh L1 medium (fig. S14). The molded samples were incubated under the same conditions as the cell cultures.

### Extrusion-based 3D bioprinting

Inks were prepared by initially dissolving alginate in cell medium. Next, CNC that act as rheological modifier were added to the alginate solution and mixed in a planetary mixer (ARE-250, Thinky, USA) three times at 2000 RPM for 5 min until the CNCs were well dispersed. After leaving the mixture overnight in a fridge, one more mixing step was performed before the addition of dinoflagellates. Another short mixing step of 1 min was carried out to ensure homogenous dispersion of cells in the hydrogel ink. The final bioink contained 1.5 wt % sodium alginate, 15 wt % of CNC, and 84.5 wt % L1 medium and had a theoretical cell density of 1 cell/μl. The bioink was transferred into a syringe, and air bubbles were removed by 2 min centrifugation at 2000*g*.

An extrusion-based desktop printer (3D Discovery, RegenHU Ltd., Switzerland) was used for 3D bioprinting of cell-laden hydrogel grids. The grid designs were created with BioCAD 1.1 (RegenHU Ltd.) and printed under pneumatic pressure in the range 0.1 to 0.5 bar. A conical polypropylene nozzle with a minimal diameter of 0.58 mm was connected to the syringe. Printed samples were cross-linked for 20 min in a 50 mM CaCl_2_ solution, followed by a washing step with DPBS. After washing, the samples were placed in L1 medium–filled petri dishes (TPP, Switzerland), closed off using parafilm, and further incubated under cultivation conditions.

### DLP 3D printing

The ink was prepared on the basis of a previously reported formulation (*15*). Briefly, 1% w/v of HAMA was first mixed in L1 medium. After full dissolution under magnetically stirring, 20% w/v Plu-DMA was added. Defoaming of the gel was accomplished by using the preset defoaming program of a planetary mixer (ARE-250, Thinky, USA) at 2200 rpm until bubble-free. The mixture was kept at 5°C under rest for at least 3 days to optimize the dispersion. While cooled in an ice bath to remain fluid, the ink was supplemented with 1 M NaOH and magnetically stirred to obtain a pH of 6. Next, quinoline yellow and LAP were added to final concentrations of 0.018 and 0.5% w/v, respectively. The ink was defoamed and was stored in a cold, dark place until further use. Concentrated dinoflagellate suspension (1 to 2% v/v) was added to the ink just before printing and gently mixed. On the basis of the luminescence of the printed gels, we found this procedure to be sufficiently gentle to keep dinoflagellates alive and metabolically active. The completed bioink was left to rest shortly at 5°C if bubbles were present and afterward added into the printer tray.

Light-based 3D printing was performed with a commercial DLP printer (Ember, Autodesk Inventor), equipped with a lamp that reaches a maximal light intensity of 22 mW cm^−2^ at 405 nm. In previous research, such printer was used to successfully manufacture living materials with resolution of 500 μm (*15*). The DLP printing process was customized to enable printing with small volumes in the range 3 to 5 ml. For this, a printing bed was redesigned to hold a 25-mm petri dish (Corning), and a printing head of 18 mm was fabricated. Furthermore, the bottom of the petri dish was coated with FEP film (fluorinated ethylene propylene copolymer film, Sigma-Aldrich) and additionally treated with rain repellent (Rain-X, Germany) to reduce adhesion to the ink. By contrast, the aluminium printhead was sandblasted to enhance adhesion. After this surface treatment procedures, the ink was added into the petri dish and was ready for printing.

The object designs were either made by Fusion360 (Autodesk) and transformed into an STL file or taken from the Thingiverse website https://www.thingiverse.com [(duck) thing:1456958 08/2024, (dinosaur) thing:6266238 11/2023, (gyroid) thing:757884 08/2024]. The STL file was transferred to Netfabb (Autodesk) for slicing and conversion into a series of black and white PNG files. The white color set the object contours, which displayed a resolution of 740 dpi (1280 px by 800 px). Printing was executed with a step size of 100 μm and an exposure time of 1 s per layer.

After printing, the object was removed with a razor blade and placed in a centrifuge tube containing L1 medium. The samples were placed on a shaker for several hours to remove excess of uncured material. Last, the yellow medium was replaced by fresh medium and the samples were incubated.

The printed objects were placed on glass slides and imaged using a camera with the settings f4.0 ISO12800 and an exposure time of 2 to 5 s. Luminescence of the cell-laden printed objects could be achieved by hitting the table onto which the samples were placed.

### Growth and viability experiments

Agar stocks were prepared as described earlier to obtain dinoflagellate-laden hydrogels with 0.5 to 3% agar. Some 150 µl of the warm gel mixtures was placed in the wells of a 96-well plate for each gel concentration (*n* = 3). After several minutes of gelling at room temperature, 100 μl of fresh L1 medium was added on top of the samples. Next, the surrounding wells were filled with sterile water, and the well-plate was closed off with parafilm to prevent drying during incubation. The samples were incubated under the same conditions used in the cell cultures. The medium was exchanged every 3rd week similarly to the other cultures, in a 3/2 fashion to account for losses by evaporation.

Cells per colony were counted utilizing an inverted microscope (*n* = 10 per well) at regular time points for 8 weeks. Confocal microscopy images and 1150 μm–sized z-stacks were recorded at different time points. Projections of the stacks were generated with FIJI (*55*) using the built-in MaxProjection function. Images and z-stacks were acquired for 20-week-old independent samples by digital and confocal microscopy.

### Mechanoluminescence of cell-laden hydrogels

The mechanoluminescent response of cell-laden hydrogels was measured by uniaxially compressing cylindrical samples under controlled strain rate. Cell-laden hydrogels with 1% agar were mold cast by adding 1.2 ml of the warm fluid gel into custom-made 10 mm–by–12 mm TEFLON molds. The molds were closed off at the top with stiff transparent paper to attain a flat surface at both sides (fig. S13). After gelling, the hydrogels were carefully demolded and transferred into the wells of 12- or 24-well plates containing 1.5 to 2 of ml fresh L1 medium. Next, the well plates were sealed off with parafilm and were left to rest for 4 to 7 days for the cells to recover from stresses and to produce luciferin. At the time shift between light and dark, the samples were taken out from the plates and left for at least 30 min in darkness before starting the tests. All tests were initiated at least 1 hour after the nighttime started.

The mechanoluminescence of the dinoflagellate-laden gels was assessed using a mechanical testing device (TA.XTplusC Texture Analyser, Stable Micro Systems, UK) equipped with a 500-g load cell. Tests were performed with a custom-made cylindrical puncher of 14-mm diameter that fits into wells of a 24-well plate. The software (Exponent Stable Micro Systems, UK) was set to compress the sample until a specified distance and return to the start position at the same rate, while recording at 200 pps. After calibration of the height, the puncher was gently placed on top of the hydrogel and the force sensor was tared. The compression distance was set on the basis of the height of the hydrogel. The contact between the puncher and the hydrogel was carefully established to prevent cell activation resulting from the suction of the water layer on the hydrogel to the puncher during the test. Because high loading rates cannot be achieved with small traveling distances, we measured the effective strain rate imposed by the mechanical testing device at different nominal loading rates (fig. S1A). The point rate for each point in time was calculated by dividing the difference in distance by the difference in time $\left(d_{x}-d_{x-1}\right)/\left(t_{x}-t_{x-1}\right)$. The maximal point rate was taken as effective rate. The effective strain rates are reported here. Video imaging was performed directly from the side or from the bottom of the sample making use of a mirror positioned at a 45° orientation. For imaging from the bottom, the sample was placed on a transparent Plexiglas surface. A sealed carton board box was placed around the mechanical testing machine to ensure dark conditions. A hole was created in the box to enable imaging using an external camera setup.

### Strain and strain rate tests

One percent w/v agar hydrogels containing dinoflagellates were produced and mechanically tested 7 days after preparation. On the day of the experiment, the liquid medium was removed from the wells 1 hour in advance of the test.

The effective strain and strain rate applied by the mechanical testing machine were assessed by compressing dinoflagellate-laden hydrogels at eight gradually increasing nominal rates (0.1 to 20 mm/s) (*n* = 4) and a predefined strain between 1 and 15%. The measured force and displacement were used to calculate the stress applied to the sample as a function of time.

To investigate the exhaustion of the bioluminescence under various strain rates, the hydrogels were subjected to an additional experiment. For this, nine samples were compressed at a rate of 2.5, 4.8, or 7.9 mm/s (*n* = 3) until a fixed strain of 10% was reached. Using these settings, 10 compression tests were executed in a continuous manner without removing the hydrogel from the mechanical testing machine.

### Long-term cell mechanoluminescence in gels with distinct stiffness

Cylindrical hydrogels with 0.5, 1, 2, and 3% w/v agar were prepared as described above to study the mechanoluminescence of dinoflagellates embedded in matrices of varying stiffness over time. The cell-loaded samples were incubated in 12-well plates (TPP, Switzerland) for 8 weeks while exchanging the medium every 3 weeks. The gels were temporarily removed from the wells after 4 days, 2 weeks, and 8 weeks to measure their mechanoluminescent response under prolonged storage conditions. For the mechanical activation of cell-laden gels with different agar concentrations, the strain was set to 12.5% and the nominal strain rates was increased from 0.5 to 10.2 mm/s during multiple cycles. In contrast to the earlier described mechanical tests, the emission was captured from the side of the hydrogels.

### Image analysis of luminescent cells

The number of luminescent dinoflagellates in mechanically loaded samples was quantified over time through image analysis in Matlab (R2021a). Luminescence was measured by extracting the intensity of the blue channel from the series of optical images acquired during mechanical loading. A mask was created around the sample to reduce background signal arising from reflections. A threshold value of 70/255 was set to distinguish the signal from noise and subtracted from the final intensity of the pixel values. For each frame, the total intensity produced by the sample was obtained by integrating the pixel intensities using the Matlab built-in function trapz. The number of luminescent cells as a function of time could be detected by running a local maxima function for each time frame. To prevent counting cells twice, the total amount of cells was calculated by applying the trapz function on the maximum projection of the stack of images (fig. S14). The total intensity and total number of luminescent cells were averaged over images acquired from replica experiments. The averages and SDs shown in the plots were obtained by the built-in mean and std functions, respectively.

### Mechanical testing of hydrogels

Hydrogels made of agar or HAMA–Plu-DMA were mechanically tested to evaluate the stress-strain response of the gels used to host the dinoflagellates. Abiotic cylindrical (12 mm by 10 mm) hydrogels with 1% agar were created following the previously described protocol (*n* = 8). Photosensitive HAMA–Plu-DMA inks were prepared as described before, leaving out the final step of the addition of the cell culture suspension. Abiotic cylindrical samples (*n* = 3) with a diameter of 10 mm and 8 mm in height were generated by DLP 3D printing. The two types of hydrogels were incubated for 4 days in conditions similar to dinoflagellate-laden gels to ensure full swelling and testing following the same protocol. Next, the agar and HAMA–Plu-DMA samples were characterized in a mechanical testing device (TA.XTplusC Texture Analyser) equipped with a 500-g load cell (movie S3). Hydrogel samples were uniaxially compressed at a strain rate of 1 mm/s in ambient conditions. The elastic modulus was determined using a linear fit in the range between 1 and 3% strain.

### Emission spectroscopy

Time-resolved mechanoluminescence spectroscopy of dinoflagellate single colonies was performed using a high-performance spectrometer (TE-cooled QE Pro, Ocean Optics) equipped with a 200-μm aperture. The integration time was set to 125 ms. Single colony measurements were enabled by using optogenetics equipment (Thorlabs), namely a 5-mm ceramic fiber optic cannula with 200-μm diameter and 0.22 numerical aperture. The low angular acceptance θ = 12.5° for incoming light facilitates spectroscopic measurements of single colonies. Hydrogels with low cell density that had been incubated for over 8 weeks, resulting in the presence of large and bright colonies, were used (movie S2). To calculate the number of emitted photons, the quantum efficiency of the spectrometer and an insertion loss of 1.0 dB at the interconnect have been taken into account (<21%). Peak deconvolution was performed using Wolfram Mathematica assuming a set of Gaussian peak profiles. The time-resolved photoluminescence data were fitted by the stretched exponential $I\left(t\right)=I_{0}\text{exp}\left[-{\left(\frac{t}{\tau }\right)}^{\alpha }\right]$ with τ = 238 ± 8 ms and α = 0.85. A stretched exponential is typically used to describe a linear superposition of a set of exponential decays (*56*).

### Testing of sensing living device

The 3D-printed device featuring pillars of distinct heights was tested using a mechanical probing device (TA.XTplusC Texture Analyser, Stable Micro Systems, UK) equipped with a 500 g load cell. The sensing device was uniaxially compressed up to 38.5% strain at a rate of 5 mm/s while recording the applied force and the bioluminescence from below. A pillar was considered to be actuated when the bioluminescence intensity surpassed a threshold of five times the SD of the background noise. The recorded image sequence was synchronized with the force measurement taking into account our experimental observation that a minimum strain of 2% is required for the actuation of embedded dinoflagellates. Under this assumption, the onset of illumination of the largest pillar p0 corresponds to a strain of 2%.

## Supplementary Materials

### The PDF file includes:

Supplementary Text Figs. S1 to S14 Table S1 Legends for movies S1 to S5 References
