# Supplementary material for: Light-based 3D printing of mechanoluminescent living gels loaded with dinoflagellates
Source: Sci Adv. 2026 Jun 5;12(23):eadz0017. doi: 10.1126/sciadv.adz0017 (PMC13240182; doi:10.1126/sciadv.adz0017)
Supplement: Supplementary file 1 — Supplementary Text Figs. S1 to S14 Table S1 Legends for movies S1 to S5 References [file sciadv.adz0017_sm.pdf]

Science Advances
AAAS

Supplementary Materials for

Light-based 3D printing of mechanoluminescent living gels loaded with dinoflagellates

Rani Boons *et al.*

Corresponding author: Gilberto Siqueira, gilberto.siqueira@empa.ch; André R. Studart, andre.studart@mat.ethz.ch

Sci. Adv. **12**, eadz0017 (2026)
DOI: 10.1126/sciadv.adz0017

The PDF file includes:

Supplementary Text
Figs. S1 to S14
Table S1
Legends for movies S1 to S5
References

Other Supplementary Material for this manuscript includes the following:

Movies S1 to S5

1. Mechanoluminescence of dinoflagellate-laden gels

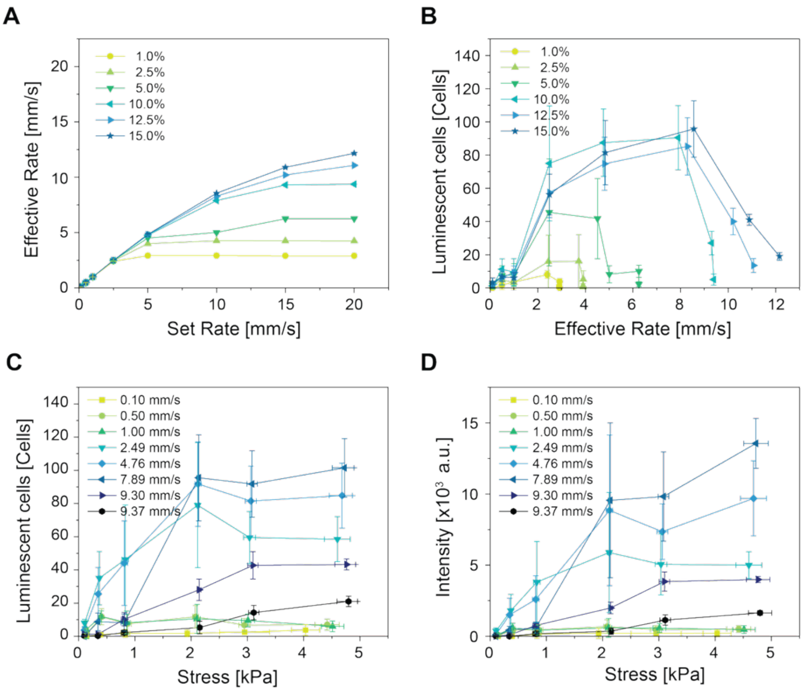

Figure S1. Mechanoluminescent response of dinoflagellate-laden hydrogels under cyclic conditions. (A) Measurements of the effective strain rates achieved upon cyclic compression of cylindrical samples (12 mm x 10 mm) at different fixed strains while ramping up the nominal (set) strain rate. The effective strain rate was calculated from the experimentally measured travelling distance and elapsed time. For low strains and high strain rates, the effective strain rates differ substantially from the nominal strain rates. (B) Effect of the effective strain rate on the number of activated cells for different maximal strains. (C,D) Effect of the applied stress on the (C) number of bioluminescent cells and (D) the total bioluminescence intensity for different effective strain rates. The bioluminescence intensity is obtained from the sum of the intensity of pixels above a threshold value integrated over time.

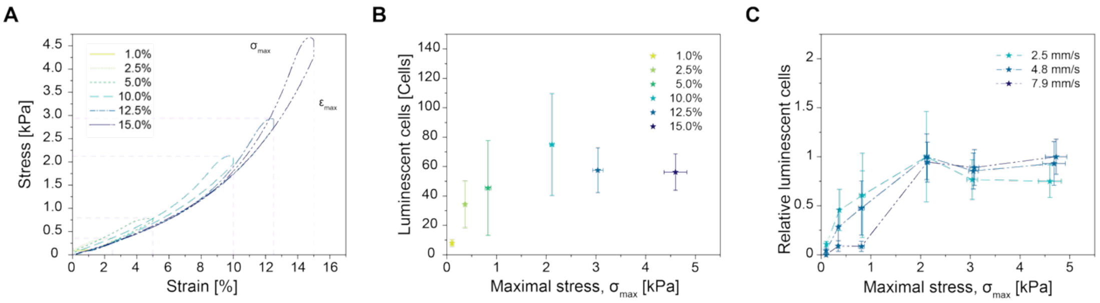

Figure S2. Mechanoluminescent response of agar gels loaded with dinoflagellates. (A) Stress-strain curves obtained for hydrogel samples during compression at 2.5 mm/s for varied maximum strains. σ_{max} indicates the maximum stress values applied. (B) The number of luminescent cells as a function of the applied maximum stress for samples tested at a strain rate of 2.5 mm/s. (C) The effect of the maximum applied stress on the relative number of luminescent cells for experiments carried out at different strain rates.

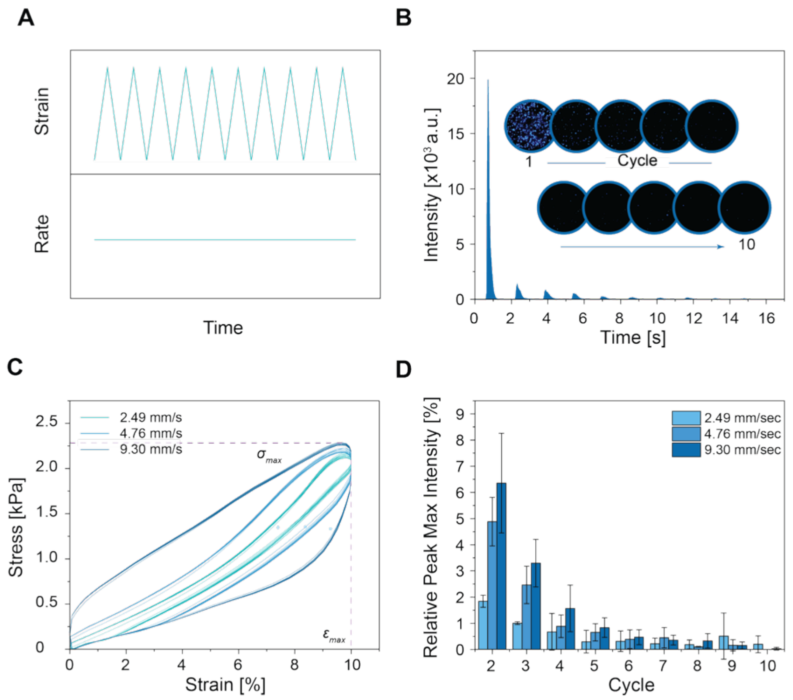

Figure S3. Exhaustion of bioluminescence acitivity under cyclic conditions. (A) Schematics displaying the strain and strain rate applied during the cyclic tests. (B) Representative measurements of the intensity of light emitted by dinoflagellate-laden hydrogels (12 mm x 10 mm) subjected to 10 mechanical loading cycles at a strain rate of 7.9 mm/s and maximal strain of 10%. The inset images show snapshots of the hydrogel at the peak intensity for each cycle. (C) Stress-strain curves measured during the cyclic tests performed at different strain rates. The fact that the stress-strain response remains the same throughout the 10 consecutive cycles (constant hysteresis) indicates that the gel shows minimal stress relaxation during the cyclic test. This implies that the decreased bioluminescence is not caused by relaxation of the hydrogel, but the result of the biological behaviour of the entrapped dinoflagellates. (D) The peak intensity of the different cycles is shown relative to the intensity measured for the first cycle (100%). The data shows a reduction in bioluminescence intensity of more than 90% after the first activation. Furthermore, a stronger decrease in bioluminescence is observed for the lower strain rates. This indicates that lower strain rates lead to the activation of most cells already in the first mechanical cycle.

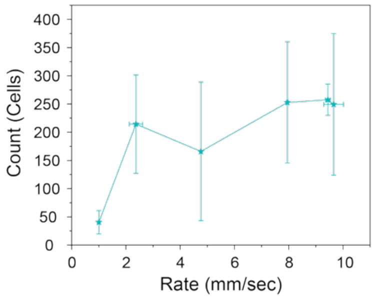

Figure S4. Mechanoluminescent response of dinoflagellate-laden hydrogels upon single uniaxial compression. The number of luminescent cells is shown as a function of the effective strain rate for cell-laden gels compressed up to a maximal strain of 10%. The data correspond mean values and their standard deviation (n=4, except for 1.0 mm/s (n=2), 4.8 mm/s (n=3) and 9.3 mm/s (n=3)). To ensure bioluminescence is not limited by the exhaustion of luciferin, independent samples were tested for each effective strain rate applied.

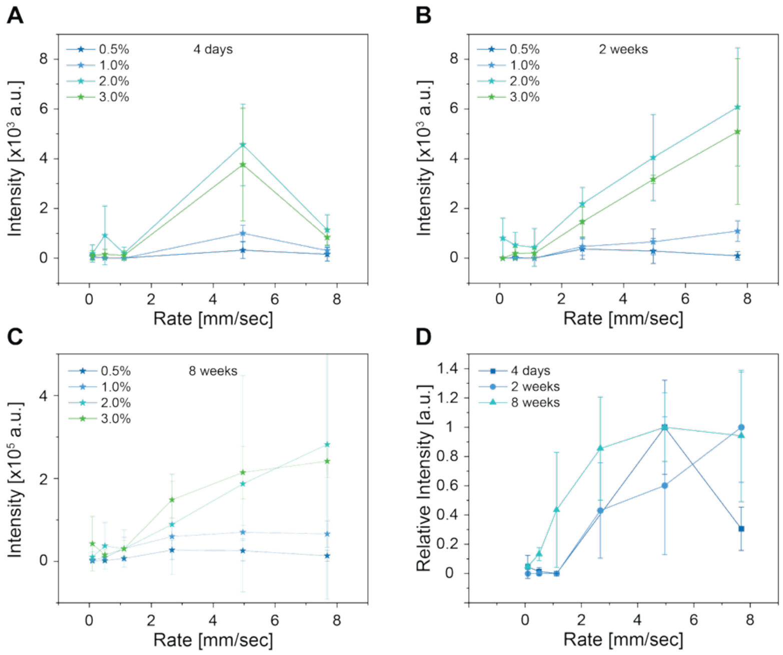

Figure S5. Mechanoluminescent response of dinoflagellates embedded in gels with different agar concentrations. (A-C) Luminescence intensity as a function of the effective strain rate for cell-laden hydrogels containing 0.5, 1.0, 2.0 and 3.0% agar after incubation for (A) 4 days, (B) 2 weeks and (C) 8 weeks (n=3). Data were obtained from cyclic compression tests with a maximal strain of 12.5%. (D) Relative luminescence intensity as a function of the effective strain rate for hydrogels containing 1% agar and incubated for different periods of time. The luminescence values are normalised to the highest mean intensity measured for each condition.

2. Modelling of mechanoluminescent response

The bioluminescence of dinoflagellate-laden hydrogels was experimentally found to depend on the effective strain rate applied in cyclic compression tests (Figure 3E). To model the change in bioluminescence observed experimentally, we propose a theoretical framework that correlates the applied strain rate ($\dot{\epsilon}$) and the number of emitted photons (n). We consider two main effects that are expected to govern the bioluminescence of dinoflagellates.

First, photoemission is known to increase with the applied strain rate $\dot{\epsilon}$ due to the viscoelastic properties of mechanosensitive biological membranes (39). Taking into account that the total number of photons n_0 is limited by the luciferin concentration, (39) we capture this effect using an ordinary differential equation (ODE) given by

$$\frac{dn}{d\dot{\epsilon}} = \alpha[n_0(\dot{\epsilon}) - n], \tag{1}$$

where α is a proportionality constant, and $n_0(\dot{\epsilon})$ is the number of available photons as function of strain rate $\dot{\epsilon}$.

Second, consecutive mechanical loading cycles lead to the exhaustion of the luciferin pool. This depletion effect decreases the total number of photons $n_0(\dot{\epsilon})$ available for emission, and can be written as

$$\frac{dn_0}{d\dot{\epsilon}} = -\beta n_0(\dot{\epsilon}). \tag{2}$$

To account for non-linear effects on n_0 and on the bioluminescence n, we introduce a non-linear strain rate $\dot{\epsilon}^{\gamma}$. This is analogous to a non-linear time in the Kohlrausch-Williams-Watts (KWW) function (57). As a result, Eq. 1 and Eq. 2 can be rewritten as,

$$\frac{d\mathrm{n}}{d\dot{\epsilon}^{\gamma}} = \alpha[n_0(\dot{\epsilon}) - n] \tag{3}$$

and

$$\frac{dn_0}{d\dot{\epsilon}^{\gamma}} = -\beta n_0(\dot{\epsilon}). \tag{4}$$

The solution of Eq. 3 is given by

$$n(\dot{\epsilon}) = n_0\left[exp\left(-\left(\frac{\dot{\epsilon}}{\epsilon_1}\right)^{\gamma}\right) - exp\left(-\left(\frac{\dot{\epsilon}}{\epsilon_2}\right)^{\tilde{\gamma}}\right)\right], \tag{5}$$

where ε_1 and ε_2 are phenomenological constants in units of strain rate, while γ and $\tilde{\gamma}$ are unitless.

For small strain rates $\dot{\epsilon}$ the photon emission is low and so is the depletion of the reservoir. In a series of cyclic loads with increasing strain rate, the emitted number of photons will first reach a maximum and then approach zero due to the exponent part of photon reservoir $exp\left(-\left(\frac{\dot{\epsilon}}{\epsilon_1}\right)^{\gamma}\right)$
Fitting Eq. 5 to the experimental data leads to the theoretical curves reported in Figure 3E. The parameters used for this fitting are shown in Table S1.

Table S1. Parameters used to fit the theoretical model (Eq. 5) to the experimental data (Figure 3E) for two dinoflagellate colonies embedded in a hydrogel subjected to cyclic mechanical loading.

	n_0	ϵ_1	γ	ϵ_2	$\tilde{\gamma}$
Colony A	742368	6.93	1.79	5.22	1.61
Colony B	170185	6.93	1.16	5.22	1.27

3. Emission spectroscopy

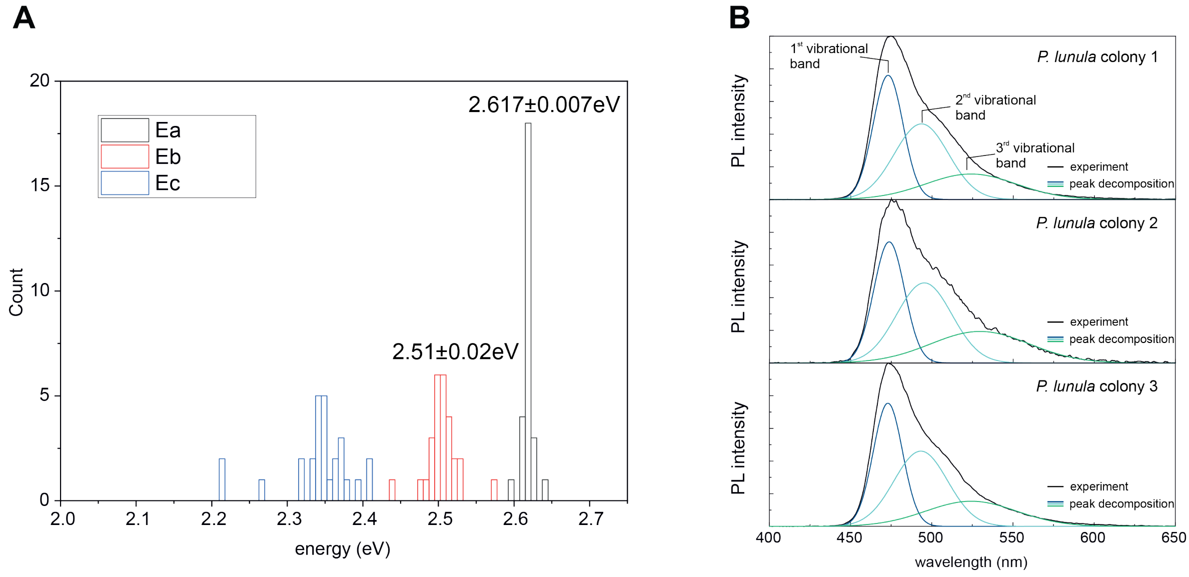

Figure S6. **Photoemission statistics obtained from emission spectroscopy experiments.** (A) Eight *P. lunula* colonies hosted in gels were subjected to compression under different strain rates. The three characteristic emission peaks are centered at E_a = 2.617 ± 0.007 eV (black), E_b = 2.51 ± 0.02 eV (red) and E_c = 2.35 ± 0.05 eV (blue). (B) Peak positions are determined from deconvoluted photoluminescence spectra exemplified for three colonies. While further research is needed to elucidate the underlying radiative decay process, the high reproducibility of the photoluminescence measured for several colonies of different sizes suggest that the emission process is not caused by a sequence of random emission events or by non-uniform stresses around the colony.

4. 3D printing of dinoflagellate-laden gels

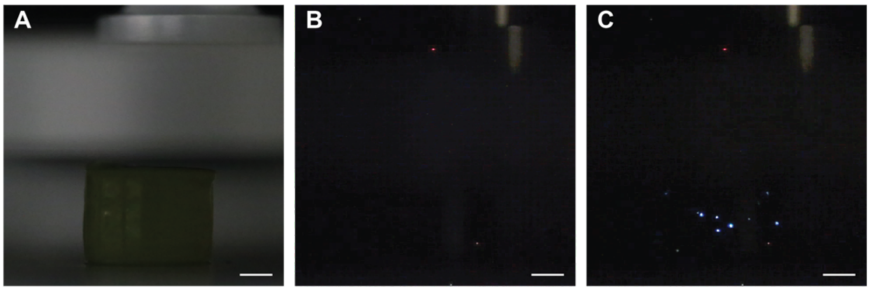

Figure S7. Mechanoluminescence of DLP-printed cylinders loaded with dinoflagellates. (A) Image of 3D printed cylindrical hydrogels containing living dinoflagellates. (B) Side-view of cylinder under compression of 20% strain reached by loading the sample with a strain rate of 0.5 mm/s. (C) Side-view of cylinder under compression of 20% strain reached by loading the sample with a strain rate of 2.5 mm/s. Scale bars = 3 mm

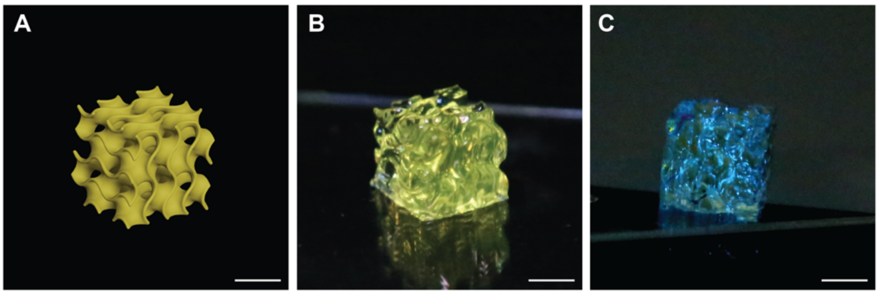

Figure S8. DLP printing of mechanoluminescent living gyroid using hydrogels loaded with dinoflagellates. (A) CAD model, (B) images of 3D printed hydrogels containing living dinoflagellates and (C) mechanoluminescent living hydrogel emitting blue light as a result of a manual impact on the table where the sample is placed. Scale bars = 5 mm.

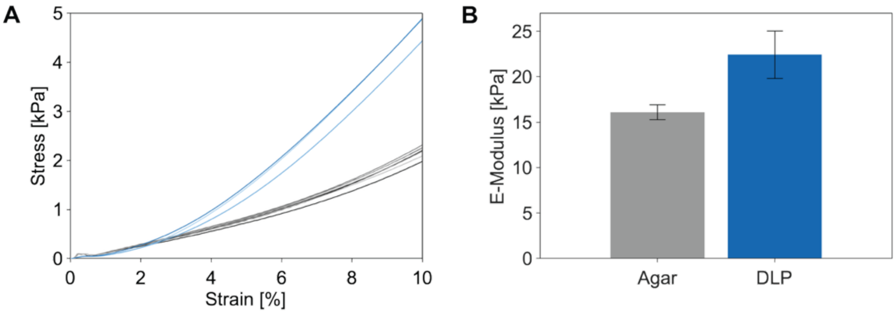

Figure S9. Mechanical properties of cell-free cylindrical hydrogels. (A) Compression stress-strain response of agar (gray, n=8) and HAMA-Plu-DMA (blue, n=3) hydrogels subjected to uniaxial compression at a strain rate of 1 mm/s. (B) Elastic moduli calculated for strains between 1% and 3%: 16 ± 1 kPa for agar and 22 ± 3 kPa for HAMA-Plu-DMA printed gels.

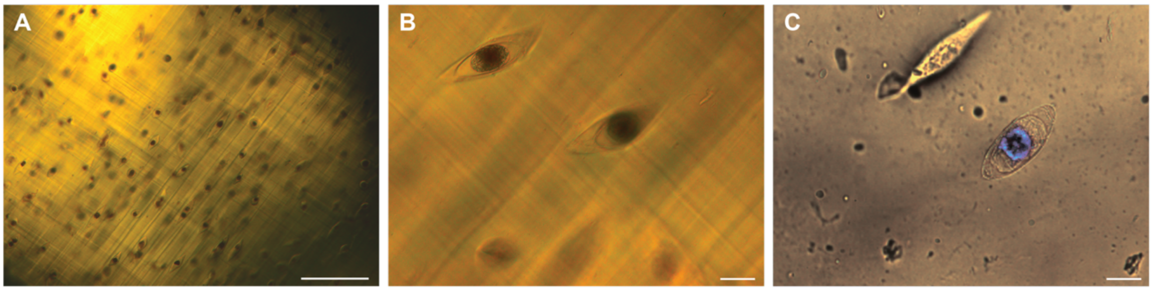

Figure S10. Optical microscopy images of *P. lunula* cells embedded in DLP-printed structures. (A,B) Dinoflagellate cells inside DLP-printed structures imaged 4 days after printing at (A) 4x and (B) 10x magnifications. The strong yellow color is given by the quinoline dye. The brown core of the cell indicates that the microorganisms are alive. (C) Mechanical stimulus on a 1.5 month old dinoflagellate-laden hydrogel triggers a bioluminescent event in the embedded cell (cyan). This demonstrates that the embedded dinoflagellates remain biologically active after a long time period. The image is composed of two microscopy images. First, an image is taken in the dark during mechanical stimulation. Under this condition, the core of the dinoflagellate cell emits blue and green bioluminescence. A second image is taken under bright light to enable visualisation of the full cell. No proliferation into colonies was noticed in this type of hydrogel. Scale bars = 500 µm (A) and 50 µm (B,C).

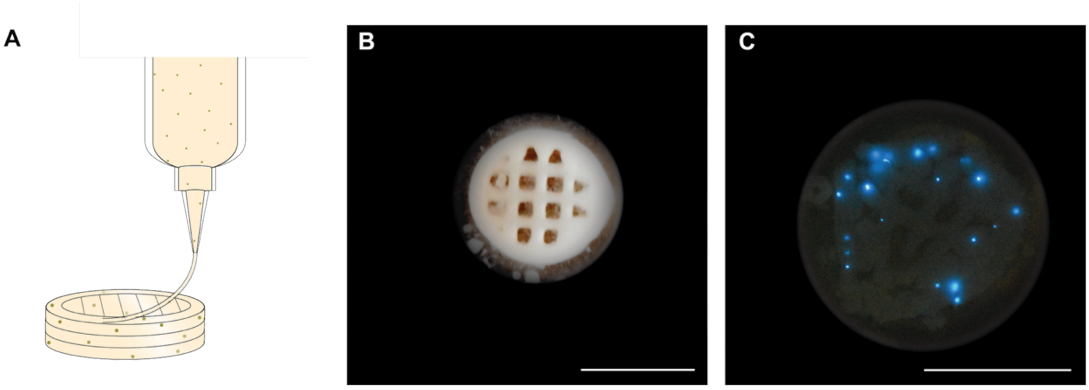

Figure S11. Complex-shaped dinoflagellate-laden hydrogel fabricated by extrusion-based 3D printing. (A) Ink containing *P. lunula* cells is loaded inside of a syringe and extruded in a layer-by-layer fashion to create a complex 3D structure. (B,C) Bottom view of the 3D printed grid structures (B) before and (C) during compression. The printed structure was compressed over 70% strain to trigger bioluminescence of the embedded cells. Scale bars = 1 cm.

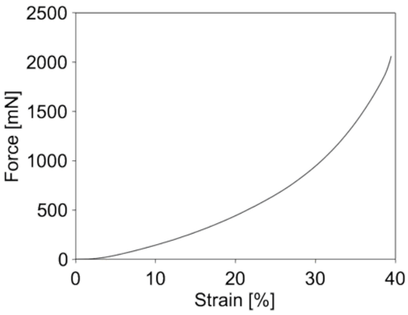

Figure S12. Mechanical response of the DLP-printed biosensor under uniaxial compression.

5. Sample preparation and cell image analysis

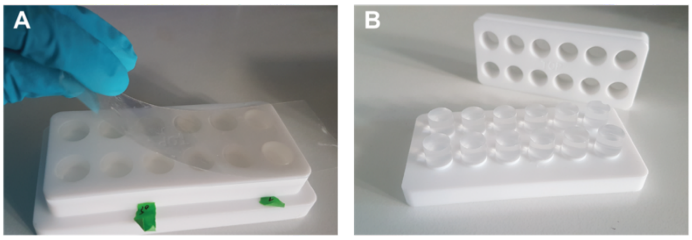

Figure S13. Molding of cylindrical hydrogels for mechanical testing. (A) Liquid agar gels cast in a mold of 10 mm height and 12 mm diameter are covered with a transparent paper. (B) Shaped agar hydrogels (1%) after opening of the dual-component mold.

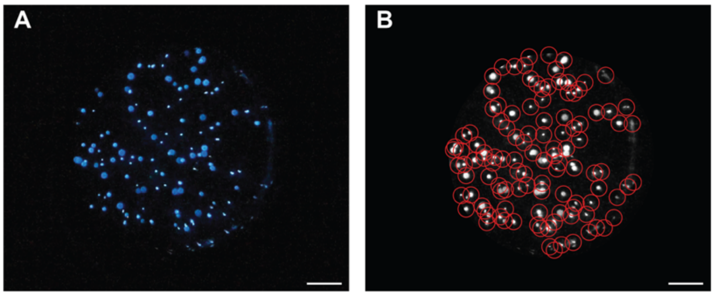

Figure S14. Image analysis of dinoflagellate-laden hydrogels during compression. (A) Maximum projection of the image series as retrieved by camera recording during compression up to 10% strain at 2.5 mm/s. (B) Red circles indicating cells detected by the blue channel after preprocessing. Scale bars = 2 cm.

6. Supplementary movies

Supplementary Movie 1. Response of dinoflagellate-laden hydrogel to multiple compression cycles. Cylindrical hydrogels (10 mm x 12 mm) were subjected to a maximum strain of 10% at 9.3 mm/s for 10 cycles.

Supplementary Movie 2. The effect of colony size on cell activation upon compression. Before the compression test, the cylindrical hydrogel (1% agar) was incubated for 8 weeks to allow the embedded cells to grow into colonies. The test was performed by applying a maximum strain of 12.5% and a strain rate of 8.3 mm/s.

Supplementary Movie 3. Elastic behaviour of 3D printed HAMA-Plu-DMA hydrogels under compression. Abiotic hydrogels with dimensions 7 mm x 9 mm (height x diameter) were compressed at 0.1 mm/s up to a maximal load of 4.5 kg, before unloading at 1 mm/s. The gel completely recovers its original height after the cycle.

Supplementary Movie 4. Bioluminescent 3D printed dinosaur-shaped hydrogel. The printed object bioluminesces while sliding on a slippery, vibrating surface.

Supplementary Movie 5. Bioluminescent 3D printed force sensor imaged from below. The pillars of the printed sensor show sequential bioluminescence when compressed.

REFERENCES

1. M. Schaffner, P. A. Rühs, F. Coulter, S. Kilcher, A. R. Studart, 3D printing of bacteria into functional complex materials. *Sci. Adv.* **3**, eaao6804 (2017).

2. P. Q. Nguyen, N. M. D. Courchesne, A. Duraj-Thatte, P. Praveschotinunt, N. S. Joshi, Engineered living materials: Prospects and challenges for using biological systems to direct the assembly of smart materials. *Adv. Mater.* **30**, e1704847 (2018).

3. X. Liu, H. Yuk, S. Lin, G. A. Parada, T.-C. Tang, E. Tham, C. de la Fuente-Nunez, T. K. Lu, X. Zhao, 3D printing of living responsive materials and devices. *Adv. Mater.* **30**, 1704821 (2018).

4. A. M. Duraj-Thatte, N. M. D. Courchesne, P. Praveschotinunt, J. Rutledge, Y. Lee, J. M. Karp, N. S. Joshi, Genetically programmable self-regenerating bacterial hydrogels. *Adv. Mater.* **31**, e1901826 (2019).

5. A. Rodrigo-Navarro, S. Sankaran, M. J. Dalby, A. del Campo, M. Salmeron-Sanchez, Engineered living biomaterials. *Nat. Rev. Mater.* **6**, 1175–1190 (2021).

6. S. Sankaran, S. Zhao, C. Muth, J. Paez, A. del Campo, Toward light-regulated living biomaterials. *Adv. Sci.* **5**, 1800383 (2018).

7. S. Molinari, R. F. Tesoriero, D. Li, S. Sridhar, R. Cai, J. Soman, K. R. Ryan, P. D. Ashby, C. M. Ajo-Franklin, A de novo matrix for macroscopic living materials from bacteria. *Nat. Commun.* **13**, 5544 (2022).

8. X. Pu, Y. Wu, J. Liu, B. Wu, 3D Bioprinting of microbial-based living materials for advanced energy and environmental applications. *Chem. Bio. Eng.* **1**, 568–592 (2024).

9. H. M. Jonkers, "Self healing concrete: A biological approach," in *Self Healing Materials: An Alternative Approach to 20 Centuries of Materials Science*, S. van der Zwaag, Ed. (Springer, 2007), pp. 195–204.

10. H. M. Jonkers, A. Thijssen, G. Muyzer, O. Copuroglu, E. Schlangen, Application of bacteria as self-healing agent for the development of sustainable concrete. *Ecol. Eng.* **36**, 230–235 (2010).

11. C. M. Heveran, S. L. Williams, J. Qiu, J. Artier, M. H. Hubler, S. M. Cook, J. C. Cameron, W. V. Srubar, Biomineralization and successive regeneration of engineered living building materials. *Matter* **2**, 481–494 (2020).

12. S. Sankaran, J. Becker, C. Wittmann, A. del Campo, Optoregulated drug release from an engineered living material: Self-replenishing drug depots for long-term, light-regulated delivery. *Small* **15**, e1804717 (2019).

13. S. Gantenbein, E. Colucci, J. Käch, E. Trachsel, F. B. Coulter, P. A. Rühs, K. Masania, A. R. Studart, Three-dimensional printing of mycelium hydrogels into living complex materials. *Nat. Mater.* **22**, 128–134 (2023).

14. A. R. Studart, K. Masania, Self-regenerating living material made of printed fungi. *Nat. Mater.* **22**, 16–17 (2023).

15. M. R. Binelli, A. Kan, L. E. A. Rozas, G. Pisaturo, N. Prakash, A. R. Studart, Complex living materials made by light-based printing of genetically programmed bacteria. *Adv. Mater.* **35**, e2207483 (2023).

16. J. Caro-Astorga, K. T. Walker, N. Herrera, K.-Y. Lee, T. Ellis, Bacterial cellulose spheroids as building blocks for 3D and patterned living materials and for regeneration. *Nat. Commun.* **12**, 5027 (2021).

17. X. Liu, Y. Yang, M. E. Inda, S. Lin, J. Wu, Y. Kim, X. Chen, D. Ma, T. K. Lu, X. Zhao, Magnetic living hydrogels for intestinal localization, retention, and diagnosis. *Adv. Funct. Mater.* **31**, 2010918 (2021).

18. L. J. Bird, E. L. Onderko, D. A. Phillips, R. L. Mickol, A. P. Malanoski, M. D. Yates, B. J. Eddie, S. M. Glaven, Engineered living conductive biofilms as functional materials. *MRS Commun.* **9**, 505–517 (2019).

19. C.-P. Tseng, F. Liu, X. Zhang, P.-C. Huang, I. Campbell, Y. Li, J. T. Atkinson, T. Terlier, C. M. Ajo-Franklin, J. J. Silberg, R. Verduzco, Solution-deposited and patternable conductive polymer thin-film electrodes for microbial bioelectronics. *Adv. Mater.* **34**, e2109442 (2022).

20. C. Li, Q. He, Y. Wang, Z. Wang, Z. Wang, R. Annapooranan, M. I. Latz, S. Cai, Highly robust and soft biohybrid mechanoluminescence for optical signaling and illumination. *Nat. Commun.* **13**, 3914 (2022).

21. C. Calvino, L. Neumann, C. Weder, S. Schrettl, Approaches to polymeric mechanochromic materials. *J. Polym. Sci. Part A Polym. Chem.* **55**, 640–652 (2017).

22. E. Poloni, A. Rafsanjani, V. Place, D. Ferretti, A. R. Studart, Stretchable soft composites with strain-induced architectured color. *Adv. Mater.* **34**, e2104874 (2022).

23. C. Larson, B. Peele, S. Li, S. Robinson, M. Totaro, L. Beccai, B. Mazzolai, R. Shepherd, Highly stretchable electroluminescent skin for optical signaling and tactile sensing. *Science* **351**, 1071–1074 (2016).

24. C. G. Schäfer, M. Gallei, J. T. Zahn, J. Engelhardt, G. P. Hellmann, M. Rehahn, Reversible light-, thermo-, and mechano-responsive elastomeric polymer opal films. *Chem. Mater.* **25**, 2309–2318 (2013).

25. C. Fajardo, M. De Donato, H. Rodulfo, G. Martinez-Rodriguez, B. Costas, J. M. Mancera, F. J. Fernandez-Acero, New perspectives related to the bioluminescent system in dinoflagellates: Pyrocystis lunula, a case study. *Int. J. Mol. Sci.* **21**, 1784 (2020).

26. B. Tesson, M. I. Latz, Mechanosensitivity of a rapid bioluminescence reporter system assessed by atomic force microscopy. *Biophys. J.* **108**, 1341–1351 (2015).

27. Y. Watanabe, Y. Tanaka, Bioluminescence-based imaging technique for pressure measurement in water. *Exp. Fluids* **51**, 225–236 (2011).

28. J. Hauslage, V. Cevik, R. Hemmersbach, Pyrocystis noctiluca represents an excellent bioassay for shear forces induced in ground-based microgravity simulators (clinostat and random positioning machine). *npj Microgravity* **3**, 12 (2017).

29. G. Han, K. Khosla, K. T. Smith, D. W. H. Ng, J. Lee, X. Ouyang, J. C. Bischof, M. C. McAlpine, 3D printed organisms enabled by aspiration-assisted adaptive strategies. *Adv. Sci.* **11**, e2404617 (2024).

30. M. R. Binelli, P. A. Rühs, G. Pisaturo, S. Leu, E. Trachsel, A. R. Studart, Living materials made by 3D printing cellulose-producing bacteria in granular gels. *Biomater. Adv.* **141**, 213095 (2022).

31. A. Saha, T. G. Johnston, R. T. Shafranek, C. J. Goodman, J. G. Zalatan, D. W. Storti, M. A. Ganter, A. Nelson, Additive manufacturing of catalytically active living materials. *ACS Appl. Mater. Interfaces* **10**, 13373–13380 (2018).

32. B. A. E. Lehner, D. T. Schmieden, A. S. Meyer, A straightforward approach for 3D bacterial printing. *ACS Synth. Biol.* **6**, 1124–1130 (2017).

33. D. T. Schmieden, S. J. Basalo Vázquez, H. Sangüesa, M. van der Does, T. Idema, A. S. Meyer, Printing of patterned, engineered *E. coli* biofilms with a low-cost 3D printer. *ACS Synth. Biol.* **7**, 1328–1337 (2018).

34. A. M. Duraj-Thatte, A. Manjula-Basavanna, J. Rutledge, J. Xia, S. Hassan, A. Sourlis, A. G. Rubio, A. Lesha, M. Zenkl, A. Kan, D. A. Weitz, Y. S. Zhang, N. S. Joshi, Programmable microbial ink for 3D printing of living materials produced from genetically engineered protein nanofibers. *Nat. Commun.* **12**, 6600 (2021).

35. C. Li, N. Schramma, Z. Wang, N. F. Qari, M. Jalaal, M. I. Latz, S. Cai, Ultrasensitive and robust mechanoluminescent living composites. *Sci. Adv.* **9**, eadi8643 (2023).

36. R. Boons, D. Gerber, R. W. Style, A. Droux, T. Zimmermann, G. Nyström, G. Siqueira, A. R. Studart, Mechanics control the proliferation of diatoms entrapped in hydrogels. *Soft Matter* **21**, 5359–5370 (2025).

37. A. Klueter, J. Trapani, F. I. Archer, S. E. McIlroy, M. A. Coffroth, Comparative growth rates of cultured marine dinoflagellates in the genus Symbiodinium and the effects of temperature and light. *PLOS ONE* **12**, e0187707 (2017).

38. E. M. Maldonado, M. I. Latz, Shear-stress dependence of dinoflagellate bioluminescence. *Biol. Bull.* **212**, 242–249 (2007).

39. M. Jalaal, N. Schramma, A. Dode, H. de Maleprade, C. Raufaste, R. E. Goldstein, Stress-induced dinoflagellate bioluminescence at the single cell level. *Phys. Rev. Lett.* **125**, 028102 (2020).

40. H. Le Ferrand, A. R. Studart, A. F. Arrieta, Filtered mechanosensing using snapping composites with embedded mechano-electrical transduction. *ACS Nano* **13**, 4752–4760 (2019).

41. J. Buck, J. F. Case, F. E. Hanson, Control of flashing in fireflies. III. Peripheral excitation. *Biol. Bull.* **125**, 251–269 (1963).

42. G. S. Timmins, F. J. Robb, C. M. Wilmot, S. K. Jackson, H. M. Swartz, Firefly flashing is controlled by gating oxygen to light-emitting cells. *J. Exp. Biol.* **204**, 2795–2801 (2001).

43. E. Y. Cherednikova, A. Y. Chikishev, O. V. Kosobokova, M. Mizuno, M. Sakai, H. Takahashi, Picosecond time-resolved absorption spectroscopy of luciferin. *Chem. Phys. Lett.* **308**, 369–372 (1999).

44. M. Valiadi, D. Iglesias-Rodriguez, Understanding bioluminescence in dinoflagellates—How far have we come? *Microorganisms* **1**, 3–25 (2013).

45. I. Navizet, "QM/MM study of bioluminescent systems" in *QM/MM Studies of Light-responsive Biological Systems*, T. Andruniów, M. Olivucci, Eds. (Springer International Publishing, 2021), pp. 227–270.

46. S. Carls-Diamante, Where is it like to be an octopus? *Front Syst. Neurosci.* **16**, 840022 (2022).

47. R. Yang, S. C. Lenaghan, M. Zhang, L. Xia, A mathematical model on the closing and opening mechanism for venus flytrap. *Plant Signal. Behav.* **5**, 968–978 (2010).

48. H. Bai, S. Li, J. Barreiros, Y. Tu, C. R. Pollock, R. F. Shepherd, Stretchable distributed fiber-optic sensors. *Science* **370**, 848–852 (2020).

49. N. Patel, R. Rana, D. Kumar, N. V. Thakor, SuperTac - tactile data super-resolution via dimensionality reduction. *Front. Robot. AI* **12**, 1552922 (2025).

50. K. Matheus, A. M. Dollar, in *2010 IEEE/RSJ International Conference on Intelligent Robots and Systems* (2010), pp. 5020–5027.

51. M. I. Latz, M. Bovard, V. VanDelinder, E. Segre, J. Rohr, A. Groisman, Bioluminescent response of individual dinoflagellate cells to hydrodynamic stress measured with millisecond resolution in a microfluidic device. *J. Exp. Biol.* **211**, 2865–2875 (2008).

52. J. Baier Leach, K. A. Bivens, C. W. Patrick Jr., C. E. Schmidt, Photocrosslinked hyaluronic acid hydrogels: Natural, biodegradable tissue engineering scaffolds. *Biotechnol. Bioeng.* **82**, 578–589 (2003).

53. K.-H. Hong, Y.-S. Jeon, J.-H. Kim, Preparation and properties of modified PHEMA hydrogels containing thermo-responsive pluronic component. *Macromolecular Res.* **17**, 26–30 (2009).

54. R. R. L. Guillard, P. E. Hargraves, Stichochrysis immobilis is a diatom, not a chrysophyte. *Phycologia* **32**, 234–236 (1993).

55. J. Schindelin, I. Arganda-Carreras, E. Frise, V. Kaynig, M. Longair, T. Pietzsch, S. Preibisch, C. Rueden, S. Saalfeld, B. Schmid, J.-Y. Tinevez, D. J. White, V. Hartenstein, K. Eliceiri, P. Tomancak, A. Cardona, Fiji: An open-source platform for biological-image analysis. *Nat. Methods* **9**, 676–682 (2012).

56. G. M. Akselrod, C. Argyropoulos, T. B. Hoang, C. Ciracì, C. Fang, J. Huang, D. R. Smith, M. H. Mikkelsen, Probing the mechanisms of large Purcell enhancement in plasmonic nanoantennas. *Nat. Photonics* **8**, 835–840 (2014).

57. A. Lukichev, Physical meaning of the stretched exponential Kohlrausch function. *Phys. Lett. A* **383**, 2983–2987 (2019).
